# Anatomical work-up of an individual with multiple muscular variants on both forearms

**DOI:** 10.1007/s12565-021-00621-y

**Published:** 2021-06-16

**Authors:** Peter S. Hagedorn, Bernhard Hirt, Thomas Shiozawa, Peter H. Neckel

**Affiliations:** Institute of Clinical Anatomy and Cell Analysis, University of Tübingen, Oesterbergstrasse 3, 72074 Tübingen, Germany

**Keywords:** Accessory muscle, Anatomical variation, Forearm musculature, *M. palmaris longus*

## Abstract

Muscular variants of the forearm are common and frequently cause neurovascular compression syndromes, especially when interfering with the compact topography of the carpal tunnel or the Canalis ulnaris. Here, we report on a male body donor with multiple muscular normal variations on both forearms. The two main findings are (1) an accessory variant muscle (AVM) on the right forearm originating from the *M. brachioradialis*, the distal radius, and the *M. flexor pollicis longus*. It spanned the wrist beneath the *Fascia antebrachia* and inserted at the proximal phalanx of the *digitus minimus*. (2) Moreover, we found a three-headed palmaris longus variant on the left arm with proximal origin tendon and a distal, trifurcated muscle belly, with separated insertions at the palmar aponeurosis, the flexor retinaculum, and, in analogy to the accessory muscle on the contralateral arm, at the base of the proximal phalanx of the *digitus minimus*. We found a considerable thickening of the left-hand median nerve right before entering the carpal tunnel indicative of a possible chronic compression syndrome adding clinical relevance to this anatomical case. We also discuss the notion that both, the AVM and the contralateral three-headed palmaris variant are developmental descendants of the *M. palmaris longus*. Additionally, we found a previously not recorded variant of the *M. palmaris brevis* on the left hand.

**Supplementary Information:**

The online version contains supplementary material available at 10.1007/s12565-021-00621-y.

## Introduction

Variants of the muscular system, especially in the forearm and wrist, are common and include various deviations, such as the absence of muscles, alternative origins, insertions, or trajectories, as well as accessory muscles (Gruber 1868; Macalister 1875; Sookur et al. 2008; Spalteholz 1939). Since they often do not cause any symptoms, most of these muscular variants remain undetected and thus a precise quantification of their prevalence remains vague. Harvie et al. (2004) found that in a collective of 58 asymptomatic test persons, 47% exhibited muscular variants of the *M. abductor digiti minimi*. Similarly, a MRI-based study detected 23 muscle variations on 42 wrists, of which in 24% there was an accessory *M. abductor digiti minimi* and in 16% the *M. palmaris longus* was missing (Zeiss and Jakab 1995). Dodds et al. (1990) detected muscular variants in 22.4% of wrists in 58 body donor documented by anatomical dissection. In contrast, in a recent report, Park (2019) found only eight anomalous muscles while conducting endoscopic carpal tunnel surgery in 973 hands. It is noteworthy, however, that this low number of variants was caused by the limited endoscopic field of view. Yet a considerably lower number of variants found within narrow and functionally important structures, such as carpal tunnel, compared to less confined topographies of e.g. the *M. palmaris longus*, would well explain the low count of symptomatic cases despite a high prevalence of muscular variants on the forearm in general.

Although most variants are incidental findings, still abnormal muscular configurations can cause neurovascular compression syndromes as reported by several authors (Al-Qattan 2004; De Smet 2002; James et al. 1987; Jeffery 1971; Lisanti et al. 2001; Ruocco et al. 1998; Salgeback 1977; Spinner et al. 1996; Zeiss and Jakab 1995). In fact, approximately 50% of muscle anomaly-caused carpal tunnel neuropathies and about 20% of ulnar tunnel compression syndromes are caused by the *M. palmaris longus* or its variants (Zeiss and Jakab 1995). Moreover, changes in the topography by missing or accessory muscles are likely to impede with surgical interventions.

In this report, we describe a previously unknown accessory muscle in the hypothenar area and wrist of the right forearm, located directly below the *Fascia antebrachii*. Additionally, we report two additional variants on the left forearm, namely a variant of the *M. palmaris brevis* with an accessory origin on the fascia of the thenar musculature as well as a *M. palmaris longus* variant with three muscular divisions inserting on the palmar aponeurosis, the flexor retinaculum and the proximal phalanx of the *digitus minimus*. Since we also found a considerably thickening of the median nerve indicative of a chronic compression syndrome, these and similar anatomical variants may have a direct clinical impact.

## Material and methods

### Body donor

The body donor was a 65-year-old Caucasian male, who died of sudden cardiac death. No morphological abnormalities such as scars or deformities were visible externally. In the latest medical record, no pre-existing conditions of the locomotor system were documented. The body donor gave his informed consent in concert with the declaration of Helsinki to use his cadaver for research purposes. The procedure was approved by the local ethical authorities (Project Nr. 237/2007 BO1).

### Fixation

The fixation was carried out by intravasal infusion via the femoral artery using an IJT-50 injection system (Thalheimer, Ellwangen, Germany). Depending on the condition of the cadaver’s vascular system, we used perfusion pressure of 0.5–1.0 bar. The fixation solution consisted of ethanol 45.5% (v/v), glycerin 23,5% (v/v), formalin 2% (v/v), and lysoformin 3,6% (v/v) in H2O.

### Photo-documentation

We photo-documented each preparation step under standardized conditions using a Nikon D300 camera with vario lens. Camera settings were standardized to 1/80 s exposure time, ISO 800 and aperture setting 16.

## Results

### Accessory wrist muscle on the right forearm

**Fig. 1:**
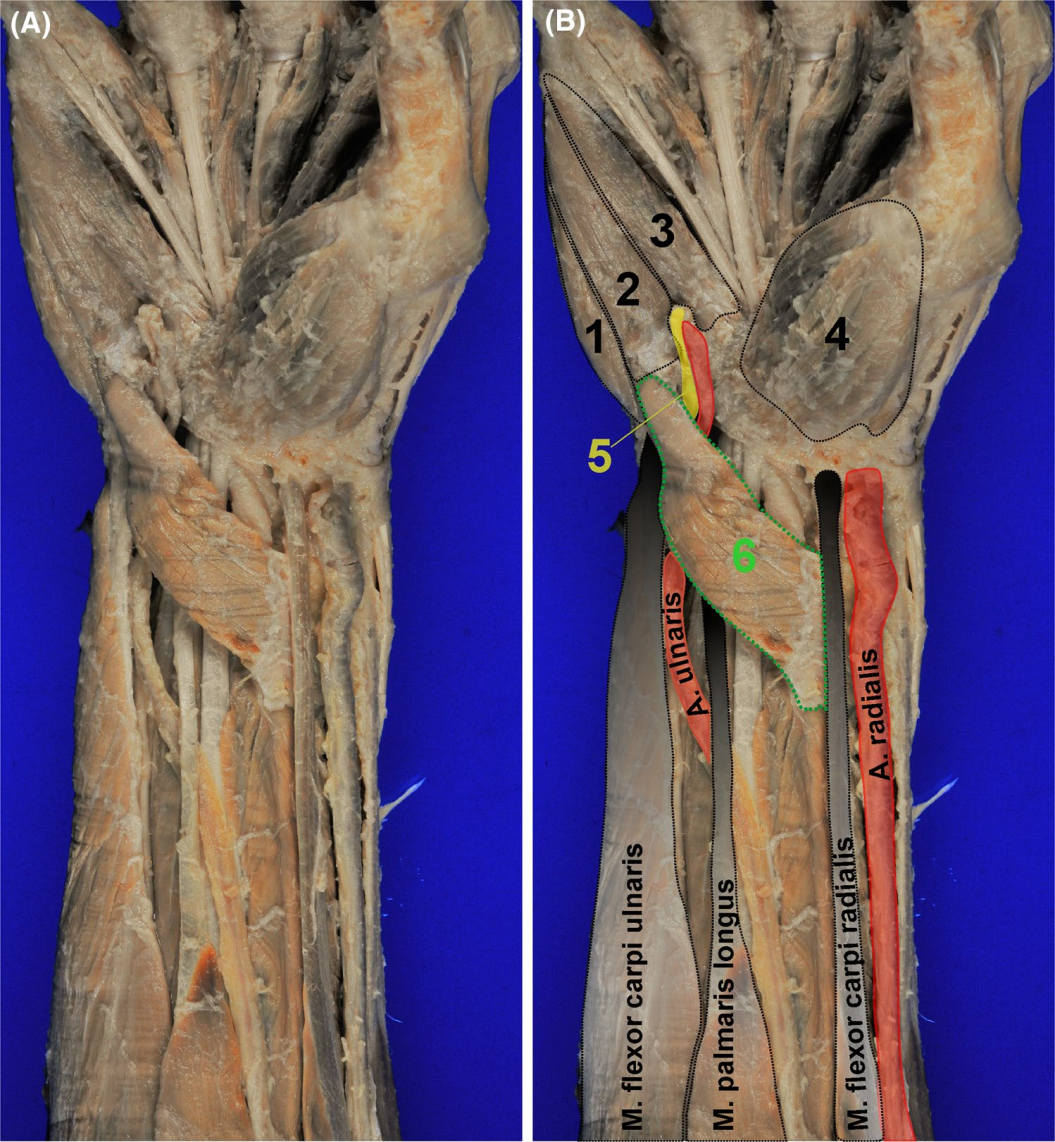
Overview of the accessory variant muscle on the right hypothanar and wrist. **A** shows an overview photograph of the accessory variant muscle (AVM) found on the right wrist and hand. In **B** the structures are annotated: 1 *M. abductor digiti minimi*; 2 *M. flexor digiti minimi brevis*; 3 *M. opponens digiti minimi*; 4 thenar musculature; 5 ulnar nerve; 6 AVM. Note that the muscle belly of the AVM lays directly below the *Fascia antebrachii* and runs over the tendon of the *M. palmaris longus*. Yet, the *M. flexor carpi radialis* and the radial artery cover the wide aponeurotic origin of the AVM

**Fig. 2:**
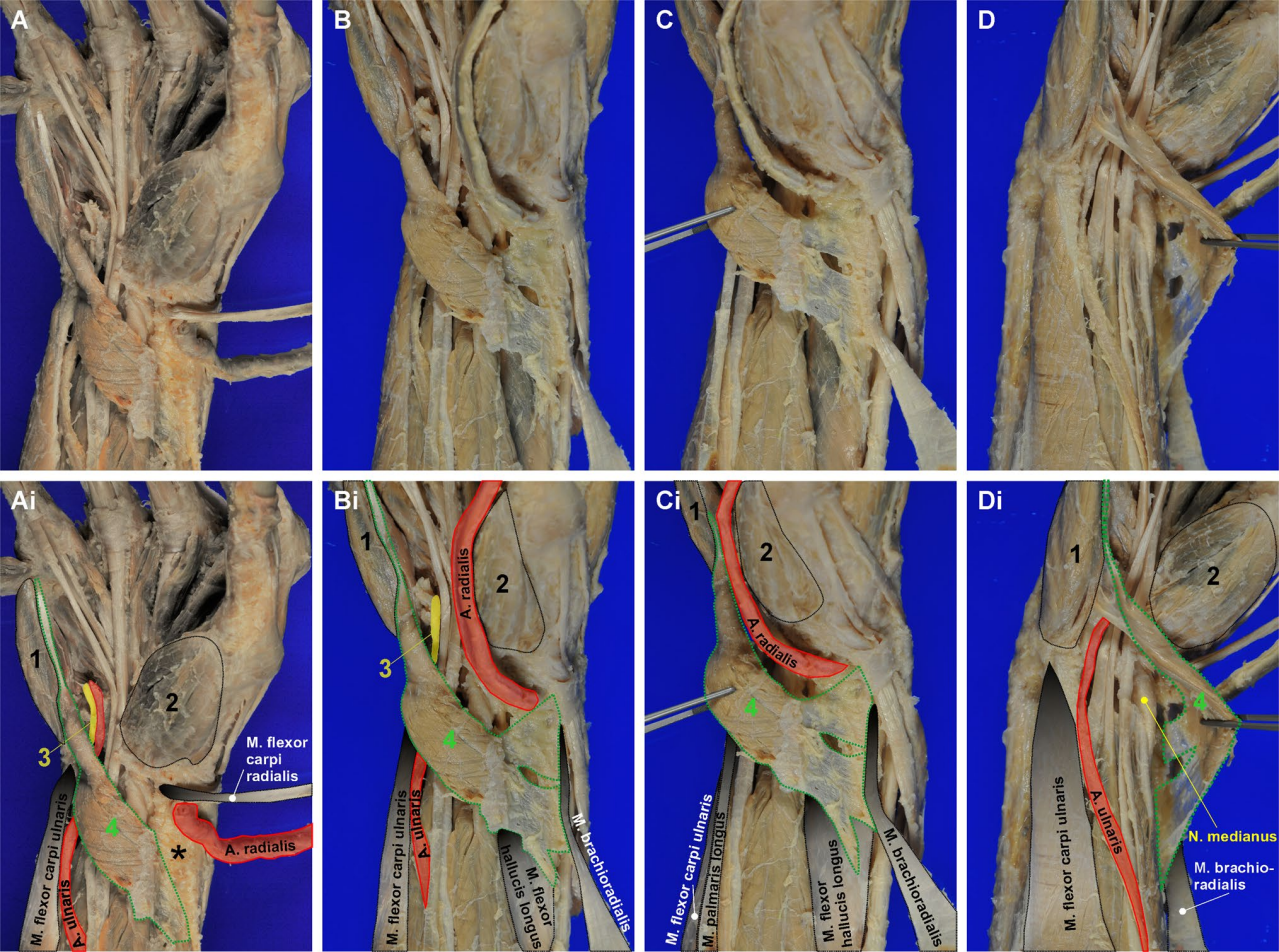
Documentation of the accessory variant muscle on the right hypothenar and wrist. **A**–**D** show photographs of the accessory variant muscle (AVM) from different angles and with full access to its origin and insertion tendons. In **Ai**–**Di** the structures are annotated: 1 M*. abductor digiti minimi*; 2 thenar musculature; 3 ulnar nerve; 4 AVM; the asterisk in A marks fatty tissue covering large parts of the aponeurotic insertion tendon of the AVM, which was removed in B-D. The AVM originates from the tendon of the *M. brachioradialis*, the belly of the *M. flexor pollicis longus*, and from the periosteum of the distal radius. The nearly cylindrical insertion tendon does not merge with other muscles of the hypothenar and inserts at the proximal ulnar aspect of the proximal phalanx of the *digitus minimus*

The accessory variant muscle (AVM) was located directly beneath the *Fascia antebrachii* on the right forearm and wrist (Figs. 1 and 2). It ran obliquely from the radial aspect of the distal palmar forearm to the hypothenar muscles. On its course, it passed underneath the radial artery and the tendon of *M. flexor carpi radialis*. Thereby, it made direct contact to the Carpal tunnel, to the ulnar tunnel, and to the ulnar artery and nerve. In its trajectory, the AVM muscle fibers curved towards the hypothenar musculature. The muscle belly narrowed to form an approximately cylindrical tendon at the proximal third of the hypothenar, which then passed between the bellies of *Mm. flexor et abductor digiti minimi* into the depth of the hypothenar musculature (Figs. 1 and 2).

We severed the radial artery, *M. abductor pollicis longus*, *M. flexor carpi radialis*, and *M. brachioradialis* to gain a better access to the AVM’s origin (Fig. 2B–D). For a better access to the insertion, we separated the *M. flexor digiti minimi* at its origin (Fig. 2A, B).

We found that the AVM exhibits a thin, but wide and tripartite origin from the tendon of the *M. brachioradialis*, with few connective tissue fibers branching of to insert at the periosteum of the distal palmar part of the radius, as well as a third origin from the belly of the *M. flexor pollicis longus*. The cylindrical tendon of the AVM inserts at the base of the proximal phalanx of the *digitus minimus* (Figs. 1 and 2).

In comparison to previously reported accessory muscles of the hypothenar and wrist, the AVM is extraordinarily large in size with a maximum thickness of 0.4 cm, a length of 7.4 cm (muscle belly), and width of 2.3 cm as measured at the belly of the muscle. Moreover, we found an even wider tripartite aponeurotic origin with a combined width of 5.5 cm. The insertion tendon is 4.5 cm long and 0.3 cm in diameter.

Due to the trajectory of the AVM and targeted pull-simulations, the presumable function was a weak abduction of the *digitus minimus* and a flexion-ulnarduction in the wrist.

### Variant of palmaris brevis on the left wrist

**Fig. 3:**
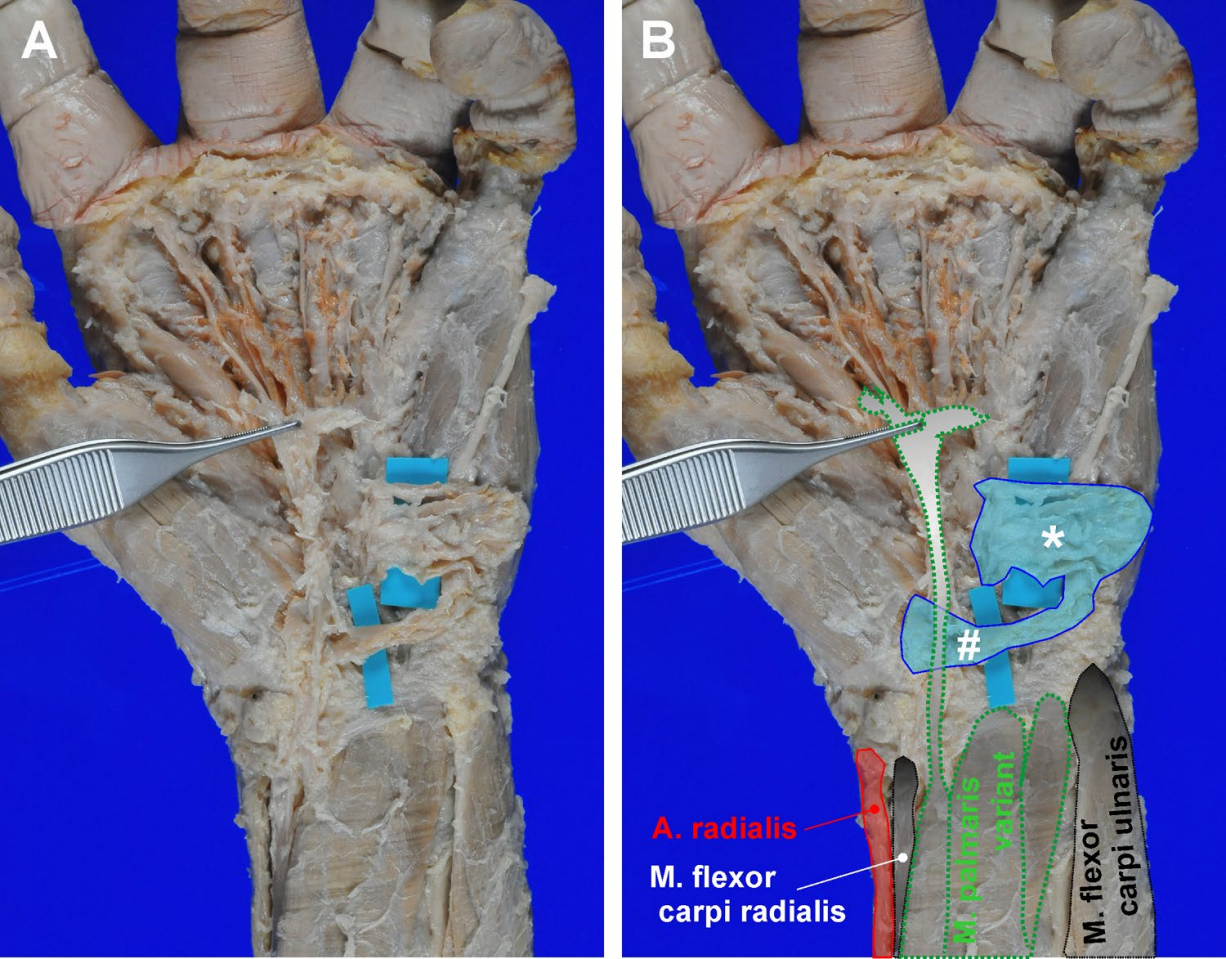
Photodocumentation of the *M. palmaris brevis* variant on the left hand. **A** shows a photograph of the *M. palmaris brevis* variant found on the left hand. **B** is the same photograph with annotated structures to help orientation. The asterisk indicates the palmaris brevis fibres regularly reported in anatomy text books, whereas # marks a bundle of accessory muscle fibres, which originate at the fascia of the thenar musculature, cross underneath the tendon of the *M. palmaris longus* to eventually merge and insert together with the regular fibres of the *M. palmaris brevis*

At the left hand, we found a variant of the *M. palmaris brevis*, consisting of larger, regularly described part with an origin at the palmar aponeurosis and an insertion just below the integument of the hypothenar region. Additionally, however, there were accessory muscle fibers originating at the fascia of the thenar musculature and running underneath the palmar aponeurosis to eventually converge and join the other palmaris brevis fibers at the regular insertion site (Fig. 3).

### *M. palmaris longus* variant on the left forearm

**Fig. 4:**
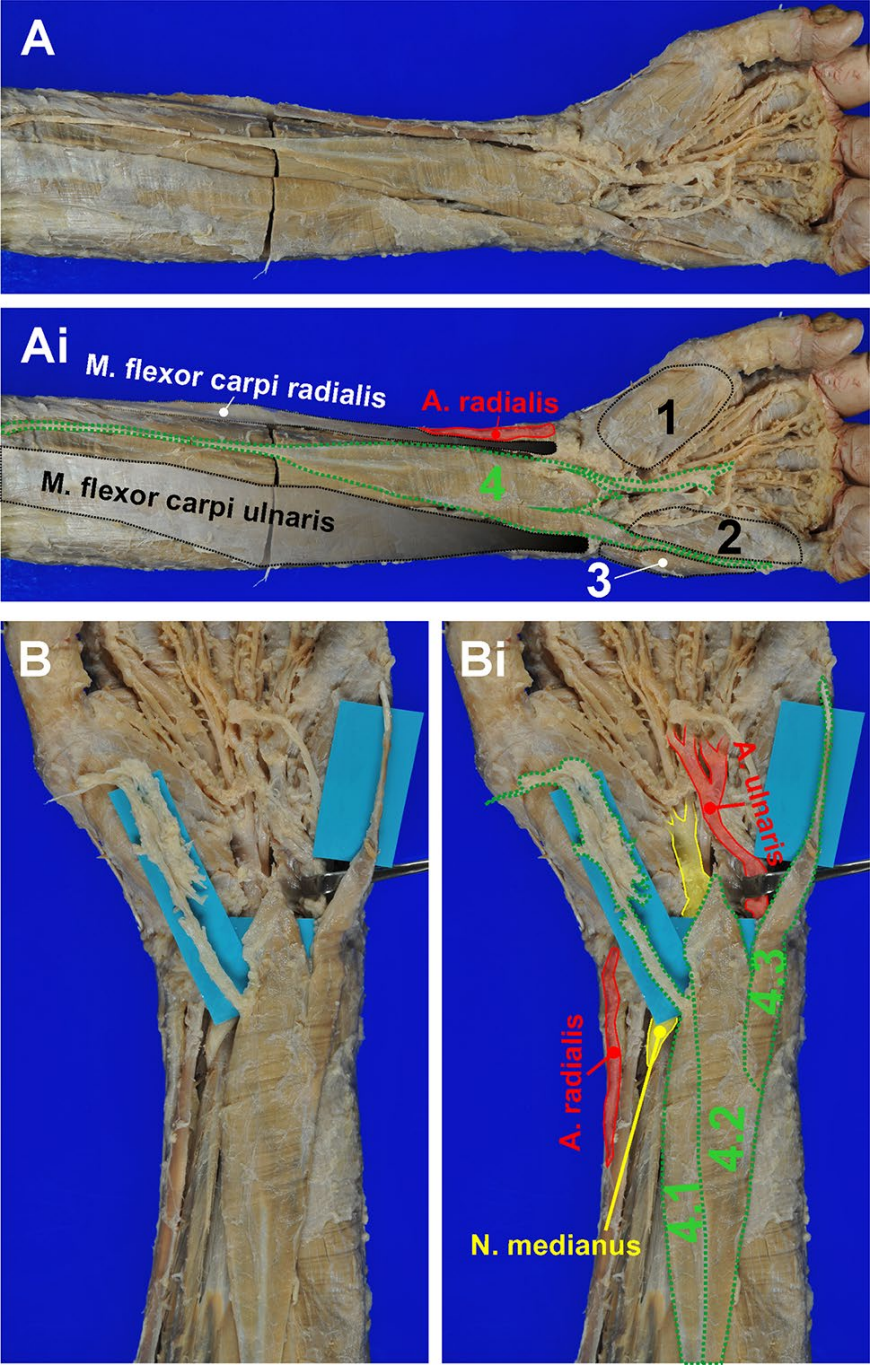
Overview of the *M. palmaris longus* variant on the left forearm and wrist. **A** and **B** show an overview and detail photograph of the *M. palmaris longus* variant (PLV) found on the left forearm. In **Ai** and **Bi** the structures are annotated: 1 thenar musculature; 2 M*. flexor digiti minimi brevis*; 3 M*. abductor digiti minimi*; 4 PLV. The PLV has a tendon of origin and large intermediate muscle belly, which in turn trifurcates and inserts in the palmar aponeurosis, the retinaculum flexorum, and the proximal phalanx of the *digitus minimus*. These insertion sites correspond to fused, but distinguishable muscle bellies (4.1–4.3). The insertion tendon of 4.1 with the attached palmar aponeurosis was displaced radially in **B** to allow a better view on the insertion site of 4.2. Note that the insertion tendon of 4.3 has the same trajectory and insertion site as the AVM on the contralateral arm

Additionally, we found a *M. palmaris longus* variant (PLV) also located on the left forearm of the body donor (Fig. 4) and originating from the medial epicondyle of the humerus with a nearly cylindrical origin tendon with 15 cm of length. Interestingly, the fleshy muscle belly was trifurcated/three-headed with three different insertion tendons. The muscle belly itself had a length of 10 cm up to its trifurcation and a maximum width of 2.6 cm at the level of the trifurcation.

The most radial insertion was a thin cylindrical tendon (diameter 0.4 cm, length 5.5 cm) fusing with the palmar aponeurosis. The middle insertion site was at the flexor retinaculum at which the muscle fibers converged to form a very short (less than 3 mm), but ribbon-like insertion tendon that directly merged with the connective tissue of the retinaculum. By following back the trajectory of the muscle fibers from these two different insertion sites, we identified two distinct muscle bellies within the PLV, which both made contact with the common origin tendon (Fig. 4B).

**Fig. 5:**
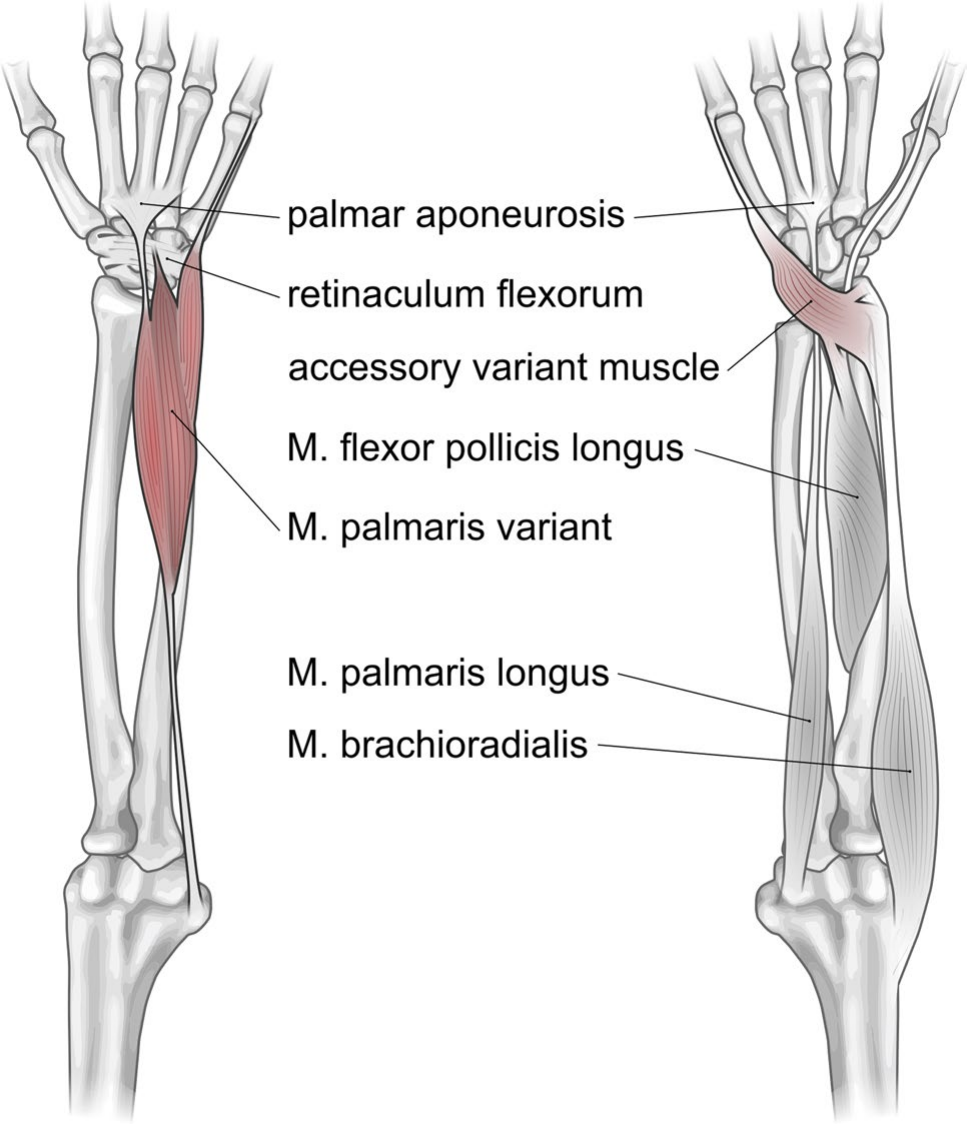
Schematic representation of the accessory variant muscle on the left wrist and the *M. palmaris longus* variant of the right forearm. Schematic drawing of the AVM and PLV (highlighted in red) based on the results of the anatomical dissection process together with other selected anatomical structures for better orientation

Intriguingly, the third fleshy muscle belly split off the distal ulnar edge of the ulnar belly of the main muscle about 3 cm of its distal end. This smaller accessory belly had a total length of 6 cm and tapered to a cylindrical 4 cm long tendon inserting at the base of the proximal phalanx of the *digitus minimus*. The insertion tendon passed between the bellies of Mm. flexor et abductor digiti minimi into the depth of the hypothenar musculature, which was equivalent to the trajectory of the insertion tendon of the AVM on the contralateral forearm (Figs. 4 and 5).

Interestingly, we found a considerable thickening of the distal median nerve right before entering the carpal tunnel on the left, but not on the right forearm (Supplementary Fig. 1), which could be indicative of a carpal tunnel syndrome (Duncan et al. 1999). However, since there were no reports of this in the medical records of the body donor, this either might have not been reported to a doctor or may have been asymptomatic.

## Discussion

Muscular variants in the distal forearm and wrist are common (Macalister 1875; Spalteholz 1939) and repeatedly have been reported to inflict neurovascular compression syndromes, especially when interfering with the confined topography in the carpal tunnel or the ulnar tunnel (De Smet 2002; Dodds et al. 1990; James et al. 1987; Jeffery 1971; Lisanti et al. 2001; Ruocco et al. 1998; Spinner et al. 1996; Zeiss and Jakab 1995). In this report, we presented the anatomical workup of a male individual with multiple, previously unknown variants on both arms. The two major findings were an accessory variant muscle on the right wrist and hypothenar as well as a variant of the *M. palmaris longus* on the left arm with one origin tendon and three separated insertion sites.

The trajectory of the insertion tendon as well as the insertion site of the AVM is similar to previously described accessory variants of the *M. abductor digiti minimi* or *M. flexor digiti minimi* (Bakinde et al. 2005; Bucher 1943; Claassen et al. 2013; Curry and Kuz 2000; Ogun et al. 2007; Ruge 1921; Saadeh and Bergman 1988; Soldado-Carrera et al. 2000; Wingerter et al. 2003; Wulle 1987); various variants have been illustrated by (Schmidt and Lanz 2003). Yet, none of the reported variants had a tripartite origin from the tendon of the *M. brachioradialis*, the belly of the *M. flexor pollicis longus*, and the periosteum of the distal radius. Astonishingly similar was an accessory hypothenar muscle reported by (Claassen et al. 2013). However, this variant did not exhibit an isolated insertion tendon but rather fused with the muscle belly of the regular *M. abductor digiti minimi*. Moreover, this variant originated from the carpal ligament, flexor retinaculum, and the palmar side of radius, yet, unlike the AVM described here, running underneath the palmaris longus tendon (Claassen et al. 2013). Other accessory *M. abductor digiti minimi* variants were found to originate at the antebrachial fascia (Saadeh and Bergman 1988; Wingerter et al. 2003; Wulle 1987) and the intercompartmental septum on the medial side of the forearm (Wahba et al. 1998) as well as at the tendon of *M. flexor carpi radialis* (Bakinde et al. 2005; Bucher 1943) or *M. palmaris longus* (Curry and Kuz 2000; Jeffery 1971; Soldado-Carrera et al. 2000). Moreover, Ogun et al. (2007) described another similar variant muscle, which originated directly from the antebrachial fascia and interestingly was found together with an ipsilateral variant of the *M. palmaris longus*. Intriguingly, Macalister (1875) described a series of accessory hypothenar muscles with comparable superficial origins at various structures in the wrist/forearm area in 1875 and concluded that these muscles are variants of a doubled *M. palmaris longus*. Further, Macalister points out that these accessory or double palmaris variants normally coexist with a regular palmaris longus muscle. Since the AVM does have a superficial trajectory running directly underneath the forearm fascia and crossing over the tendon of the regularly configured M. palmaris longus, it is conceivable that the AVM presented here is a developmental derivative of the *M. palmaris longus* as intended by Macalister. A recent study on cleared human fetuses revealed that the palmaris longus muscle arises from a common primordium with *M. flexor carpi ulnaris* and other flexors during the gestational week 7 (Diogo et al. 2019). It is conceivable that the AVM also descents from this common anlage. Furthermore, it is noteworthy that a developmental derivation from hypothenar primordia or the brachioradialis muscle, as supported by the AVM’s insertion or origin, respectively, cannot be ruled out by our topographic analysis.

The *M. palmaris longus* is arguably the most variable muscle in the human body (Gruber 1868; Spalteholz 1939) with descriptions of its variations, or its absence, respectively, dating back to the sixteenth century (Andreas Vesalius’ *De humani corporis fabrica* and Renaldus Columbus’ *De re anatomica*; see also (Brinkman and Hage 2016). Besides the absence of the muscle, which is the most common anomaly (Reimann et al. 1944; Schwalbe and Pfitzner 1894), various variation have been described over the centuries (Macalister 1875), including the location and form of its fleshy part, aberrancy of attachment at its origin or insertion, duplication and triplication, accessory slips, and replacing elements of a similar form or position (Bergman et al.; Georgiev et al. 2017; Gruber 1868; Schmidt and Lanz 2003). Concerning the PLV presented here, it is most interesting that while several authors described bifurcated or trifurcated insertion tendons sent out by a proximal muscle belly, only a few have reported multiple muscle heads. In fact, we were able to find only three other reports on three-headed palmaris longus variants, however, with differing insertion sites (Acikel et al. 2007; Natsis et al. 2007; Yildiz et al. 2000). Intriguingly, the presence of accessory *M. abductor digiti minimi* variants, both detached and fused with reversed or intermediate palmaris variations, have been described previously (Georgiev and Jelev 2009; Gruber 1868; Macalister 1875; Rauber and Kopsch 1998). Moreover, the trajectory of the ulnar head of the PLV described here is identical to the insertion of the AVM found on the contralateral arm, thereby additionally supporting the notion that the variants on both arms are variations of the palmaris longus muscle. Alternatively, the three-headed palmaris variation we found could also be the product of a bi-headed palmaris longus fused with an accessory *M. abductor digiti minimi*.

Numerous authors have reported neurovascular compression syndromes caused by muscular variations or accessory muscles (De Smet 2002; Dodds et al. 1990; James et al. 1987; Jeffery 1971; Lisanti et al. 2001; Ruocco et al. 1998; Spinner et al. 1996; Zeiss and Jakab 1995). Interestingly, two of the previously mentioned three-headed palmaris variations were causative for median nerve compression symptoms and thus were discovered in surgery (Acikel et al. 2007; Yildiz et al. 2000). In the present case, we found a considerable thickening in the median nerve directly before entering the carpal tunnel on the left side, which arguably indicates a chronic compression caused by the PLV (Duncan et al. 1999). Yet, it is noteworthy that we did not find any report on median nerve compression symptoms in the medical records of the body donor suggesting either that the presence of the PLV was asymptomatic or that the body donor never sought medical advice on his symptoms. Still, it is highly conceivable that variants in close proximity to important anatomical structures can cause symptoms of nerve and artery compression and additionally hamper in-surgery orientation with potential complications.

## Supplementary Information

Below is the link to the electronic supplementary material.

Supplementary Figure 1 Photodocumentation of the median nerve on the left wrist. Photograph of the left wrist with opened retinaculum flexorum for better view of the median nerve. The median nerve (asterisk) exhibited a considerable increase in volume right before entering the carpal tunnel, which reportedly is indicative of a chronic compression syndrome (TIF 15539 KB)

## Data Availability

Not applicable.
