# Supplementary figures and images for: Anatomical work-up of an individual with multiple muscular variants on both forearms
Source: Anat Sci Int. 2021 Jun 16;96(4):556–63. doi: 10.1007/s12565-021-00621-y (PMC8338858; doi:10.1007/s12565-021-00621-y)

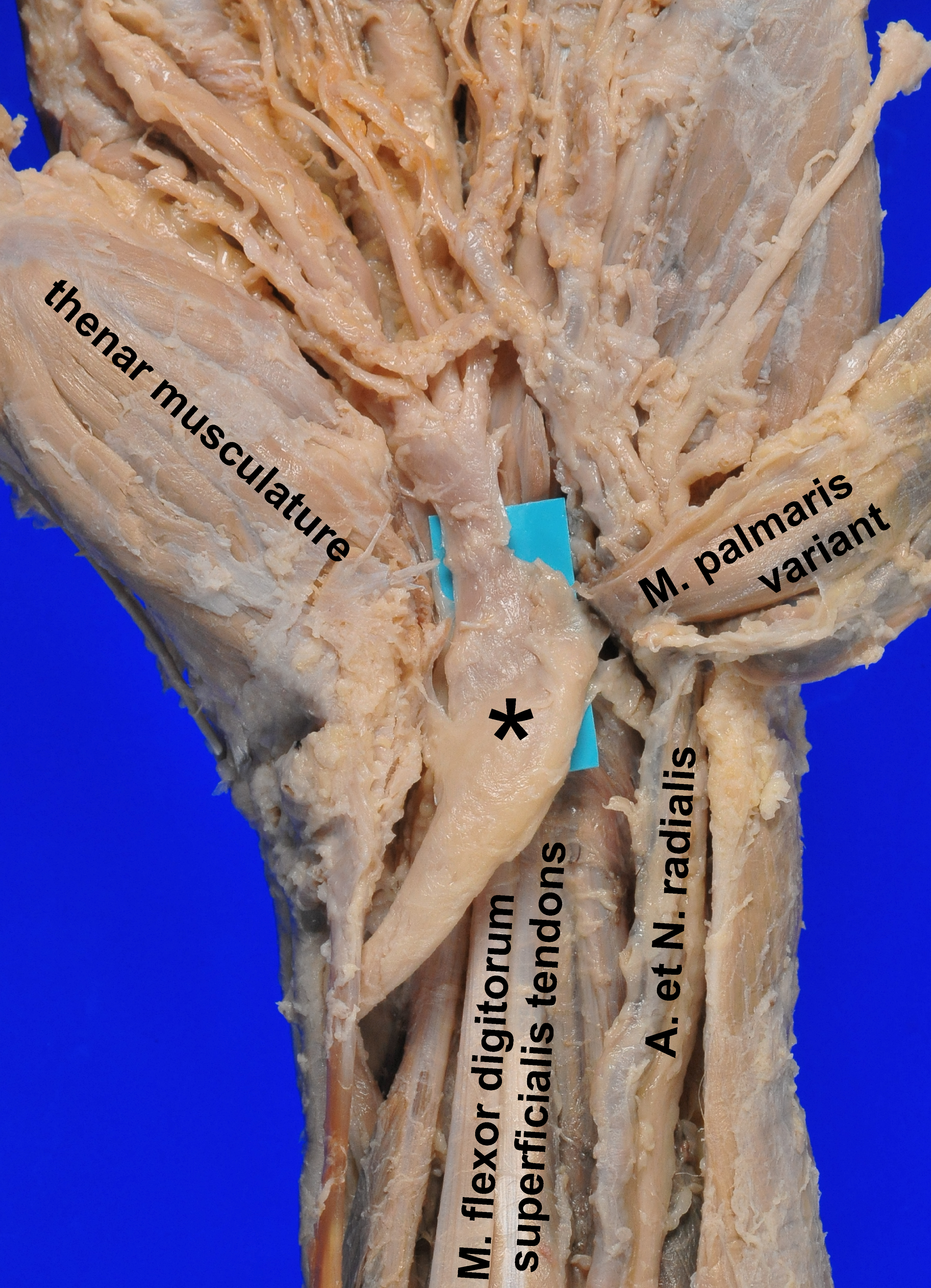

Supplement: Supplementary file 1 — Supplementary Figure 1 Photodocumentation of the median nerve on the left wrist. Photograph of the left wrist with opened retinaculum flexorum for better view of the median nerve. The median nerve (asterisk) exhibited a considerable increase in volume right before entering the carpal tunnel, which reportedly is indicative of a chronic compression syndrome (TIF 15539 KB) [file 12565_2021_621_MOESM1_ESM.tif]
